# Dual-channel microcantilever heaters for volatile organic compound detection and mixture analysis

**DOI:** 10.1038/srep28735

**Published:** 2016-07-06

**Authors:** Ifat Jahangir, Goutam Koley

**Affiliations:** 1 Department of Electrical Engineering, University of South Carolina, Columbia, SC 29208, USA; 2 Department of Electrical and Computer Engineering, Clemson University, Clemson, SC 29634, USA

## Abstract

We report on novel microcantilever heater sensors with separate AlGaN/GaN heterostructure based heater and sensor channels to perform advanced volatile organic compound (VOC) detection and mixture analysis. Operating without any surface functionalization or treatment, these microcantilevers utilize the strong surface polarization of AlGaN, as well as the unique heater and sensor channel geometries, to perform selective detection of analytes based on their latent heat of evaporation and molecular dipole moment over a wide concentration range with sub-ppm detection limit. The dual-channel microcantilevers have demonstrated much superior sensing behavior compared to the single-channel ones, with the capability to not only identify individual VOCs with much higher specificity, but also uniquely detect them in a generic multi-component mixture of VOCs. In addition, utilizing two different dual channel configurations and sensing modalities, we have been able to quantitatively determine individual analyte concentration in a VOC mixture. An algorithm for complete mixture analysis, with unique identification of components and accurate determination of their concentration, has been presented based on simultaneous operation of an array of these microcantilever heaters in multiple sensing modalities.

Volatile organic compounds (VOCs), ubiquitous in household and industrial air in varying concentration and composition, are often associated with health hazards, especially many respiratory diseases for children and adults^1^,^2^,^3^. Due to the large number VOCs commonly found in commercial and domestic products, it is a formidable task to develop an affordable technology that can detect even the most common ones accurately over a wide range of concentration with high specificity. The popular techniques for VOC detection include photo-ionization detectors (PIDs)^4^, suspended hot bead pellistors^5^, heated metal oxides^6^,^7^,^8^,^9^, functionalized quartz crystal microbalance (QCM) technique^10^,^11^,^12^,^13^,^14^,^15^,^16^, ionic liquid based techniques^17^, gas chromatography and mass spectroscopic techniques^18^. The detection methodology using PIDs is based on high-energy photon (typically >10.5 eV) induced ion generation which has poor selectivity; other technologies utilizing the principle of selective optical absorption include vacuum ultraviolet (VUV) detection^19^ and holographic detection^20^, to name a few. On the other hand, hot bead pellistors make use of the exothermic reaction (from auto-ignition of VOCs) to produce a change in resistance. Heated metal oxide (i.e. TiO_2_ or SnO_2_) based sensing also relies upon a change is resistance^21^, but catalytic oxidation keeps the operating temperature below the auto-ignition temperature of the VOCs. None of these techniques are selective. QCM based techniques are based on the shift in resonance frequency due to the change of mass caused by absorption or desorption of chemical species. This method achieves selectivity through functionalization, such as polymer coatings^10^, molecular imprinting^11^, organic/inorganic hybrid thin film coating^12^, surfactant coated carbon nanotube networks^13^, etc. These functionalization techniques can offer limited selectivity to a particular group of chemicals but are not widely applicable. A major issue with all these sensors based on functionalization is the deterioration of the functionalization layer over time, which can make them quite unreliable. In contrast, the physical property based techniques such as, mass spectroscopy and gas chromatography can offer high selectivity and sensitivity, but these systems require sophisticated measurement instrument with higher power demand. In summary, while each of these techniques offers a few desirable features of an ideal and portable VOC detection system, currently there is no existing technology that is at the same time portable, energy efficient and capable of rapid, sensitive and unique detection of VOCs, especially in multi-analyte mixtures, without any functionalization layer.

Microcantilevers are excellent candidates for molecular sensing which stems from their high sensitivity to various physical parameter changes induced by analyte molecules^22^,^23^,^24^,^25^,^26^,^27^,^28^,^29^,^30^,^31^,^32^,^33^. While they find many applications where their resonance properties are utilized, they are also used as microcantilever heaters which are extremely sensitive to changes in thermal parameters^34^,^35^,^36^,^37^,^38^,^39^,^40^, and therefore have been widely utilized for calorimetry^35^, thermal nanotopography^36^ and thermal conductivity measurements^37^. Owing to the small area of the microcantilever that needs to be heated (i.e. the tip of the microcantilever), they are suitable for high temperature operation with reduced power consumption. However, it is often very challenging to achieve repeatable and reliable functionalization of a microcantilever surface, which hinders the development of practical applications of microcantilever based sensors. This is a major setback since without functionalization, microcantilevers (typically made of Si) just by themselves are not particularly sensitive toward a specific analyte. Thus, only a handful of studies utilizing uncoated microcantilevers to perform unique molecular detection have been reported so far^30^,^41^,^42^. Detection schemes demonstrated in these studies are generally based on changes in physical properties of the media surrounding the cantilever (i.e. viscosity^42^, thermal conductivity^41^, or the deflagration temperature^41^ associated with the analyte). These techniques are generally incapable of determining the constituents of a mixture of unknown analytes, and achieving selectivity and good sensitivity together has been quite elusive especially when the analytes are diluted or have similar physical properties which are two common traits of the VOCs found in the indoor atmosphere.

III-Nitride heterojunction (especially AlGaN/GaN) based microcantilevers are very promising for realizing these microscale heaters, especially due to the presence of high carrier (electron) density in close proximity to the surface^43^, which allows for highly efficient surface heating. In addition, strong spontaneous polarization of III-Nitride surfaces allows these heaters to interact better with VOCs, which are typically strongly polar in nature. Finally, the wide band gap of the AlGaN/GaN heterojunction system promotes chemical inertness, which allows the cantilever heaters to operate at high temperature and in harsh environment without any noticeable degradation over a long time. Apart from these salient features, commercial availability of high quality AlGaN/GaN heterojunciton epilayers on Si and the existence of a straightforward fabrication process have also played vital roles in gaining popularity for this material system^28^,^44^,^45^.

In our previous work^34^,^46^, we demonstrated a triangular microcantilever heater (TMH) with high selectivity to many different types of VOCs, using a very unique mechanism never reported before. The present work introduces dual channel microcantilever heaters where two conducting channels are constructed on the same cantilever to combine multiple modes for detecting VOCs with higher degree of specificity and selectivity. These dual channel microcantilever heaters (DC-MH) combine the modalities offered by the triangular microcantilever heater (TMH) with novel sensing mechanisms based on heat transfer between the channels interacting with the VOC vapors. Using these cantilevers in an array, it is possible to detect each compound in a mixture of VOCs along with its approximate concentration. This allows selective detection of a vast number of VOCs containing one or more functional groups; such as alcohols, ethers, ketones, alkanes (straight-chain and cyclic), aromatic, alkyl halides, amides, carboxylic acid compounds and their derivatives. The detection occurs below the auto-ignition temperature of the analytes and the responses have been found to strongly correlate with their latent heat of evaporation and dipole moment just like the TMH^34^, with much higher sensitivity, selectivity and also with a way to estimate the concentration. While a lower limit of detection of 5 ppm have been experimentally established, actual limit is expected to be lower than 1 ppm limited by the noise in these devices.

## Results and Discussion

**Figure 1:**
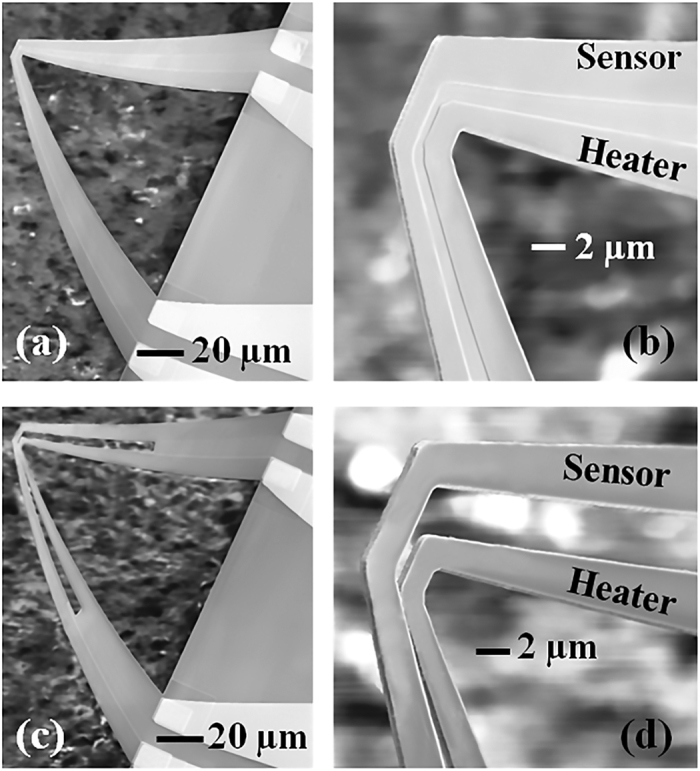
(**a,b**) SEM images of a monolithic tip dual channel microcantilever heater (MDC-MH) with sensor and heater channels marked in (**b**). (**c,d**) Split tip dual channel microcantilever heater (SDC-MH) with sensor and heater channels marked in (**d**).

The SEM images in Fig. 1 show the dual channel microcantilever heaters that were fabricated and characterized in this study. Figure 1(a,b) show a cantilever with two parallel channels isolated by etching the AlGaN mesa in between them. As marked in Fig. 1(b), the inner channel is called the *heater channel* as this is used to heat up the tip region, affecting the electrical conductivity of the outer channel marked as *sensor channel*. Since both channels are physically connected by the GaN layer, we call it *monolithic tip dual channel microcantilever heater* (MDC-MH). Figure 1(c,d) show another variation of the dual channel microcantilever heater named as *split tip dual channel microcantilever heater* (SDC-MH), where the sensor and heater channels are isolated near the tip region. For this device, conduction/convection through air or any other gaseous species is the primary mode of heat transfer from one channel to another.

**Figure 2:**
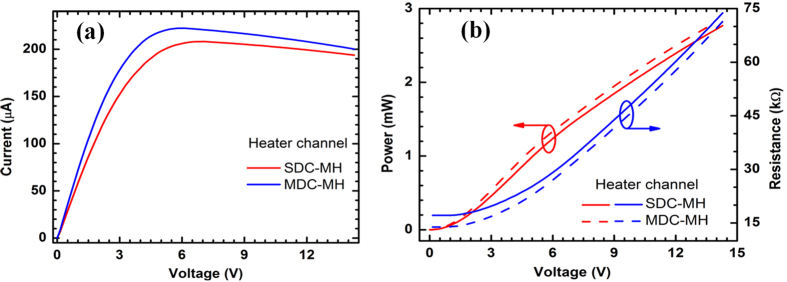
(**a**) I–V characteristics of the heater channels of monolithic and split tip dual channel microcantilever heaters (MDC/SDC-MH). Sensor channels also exhibit very similar I-V characteristics (please refer to Figure S1 of the Supporting Information). (**b**) Variation in consumed power and channel resistance with bias voltage obtained from the I-V characteristics in (**a**).

Since both types of cantilevers have gradually tapered tips, power dissipation under a voltage bias is mostly concentrated at those areas, causing rapid heat generation even with a small bias voltage. The current-voltage (I-V) characteristics of the heater channels of the MDC-MH and SDC-MH are shown in Fig. 2(a) where strong non-linearity is observed. The current is observed to decrease after ~5 V, which can be attributed to self-heating along with other commonly observed current-limiting effects. The slight variation in the I-V curves can be attributed to the process variation as well as the geometrical differences between the two types of cantilevers. The sensor channels also exhibit very similar I-V characteristics (please refer to Figure S1 of the Supporting Information); however, they are normally operated at several tens or hundreds of mV of constant dc bias only, which falls well inside the linear region. Figure 2(b) shows how power and resistance changes for the heater channel of either device as the voltage bias varies. Here we see an almost 270–370% change in resistance (from 15 to 70 kΩ for SDC-MH and from 20 to 75 kΩ for MDC-MH) as the bias changes from 0 to 14 V, with a maximum power dissipation below 3 mW. This change in channel resistance plays a pivotal role in the operation of these devices as it enhances the response of the devices by various degrees at different bias voltages.

**Figure 3:**
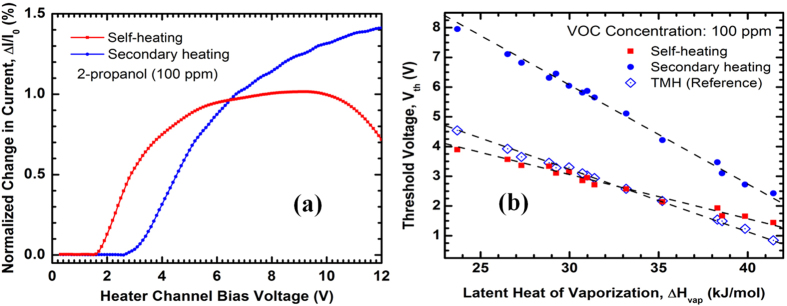
(**a**) Composite plots of normalized change in current (%) with change in voltage for 2-propanol at 100 ppm molar concentration, obtained from the outer channel (sensor channel) and inner channel (heater channel) of a monolithic tip dual channel microcantilever heater (MDC-MH). The sensor channel had a dc bias of 100 mV. (**b**) Linear relation between threshold voltage of sensing (V_th_) and latent heat of evaporation (ΔH_vap_) for different VOCs at 100 ppm molar concentration obtained from each channel of the same MDC-MH device. The response from a reference TMH device with similar dimensions and device parameters is also presented for comparison.

The two parallel channels of MDC-MH can be biased separately and the GaN section between them allows heat transfer between them while maintaining electrical isolation. While it is possible to apply various biasing configurations, in the present work we focused on one particular configuration that produced several useful observations. We applied a low dc bias of 100 mV across the outer channel (*sensor channel*), while sweeping the voltage from 0 to 10 V across the inner channel (*heater channel*) at 1 kHz sampling rate and 100 mV resolution. We measured the normalized change in current on both channels with and without the VOCs and identified changes in currents on both channels in presence of VOCs as shown in Fig. 3(a) for 100 ppm molar concentration of 2-propanol. Interestingly, ΔI/I_0_ does not start to rise until bias voltage exceeds a particular voltage, which is different for each channel. We can explain this observation for the heater channel using our prior report on the TMH^34^. As explained in that proposition, the VOC molecules tend to conglomerate in the air and get drawn to the vicinity of the heated tip region of the heater channel due to convection, which then absorb the latent heat from the tip to evaporate again. This results in a drop of temperature at the tip region exhibiting a change in resistance and current. The other parts of the cantilever do not take part in this event due to their lower temperature and also have much lower resistance compared to the tip due to their wider cross sectional areas. However, they serve as a negative feedback system to compensate for the change in tip resistance up to a certain voltage above which they can no longer keep the total resistance constant, this transition is marked by the threshold voltage (V_th_). As we kept on increasing the voltage above V_th_, the slope of ΔI/I_0_ initially decreased until it became significantly negative at or above 10 V, indicating a sharp decrease in the device response. The drop of device response after 10 V could be attributed to two main reasons–firstly the tip created an extended hot air domain around it which evaporated the molecules before it could come close to the device surface, thus reducing the magnitude of current change. Secondly, other means of heat transfer dominated over the heat loss due to VOC evaporation, reducing its effect on the device response although the temperature of the tip region kept on increasing.

Due to the very small dimension of the tip region and the significant amount of bending present along the arms of the cantilever, it was not possible to measure the temperature of the apex of the cantilevers studied in this work using infrared thermal microscopy or Raman spectroscopy, as was done in our previous work on the TMH with a larger and flatter geometry^34^. From that work (based on imaging and spectroscopic data), we inferred that the temperature of the heater channel would not exceed 200 °C for the threshold response of any analyte in self-heating mode. Based on those results we expect the channel temperature of the current microcantilever heaters to be around 200 °C for a bias voltage of 5 V applied across the heater channel. The sensor channel, on the other hand, is not expected to generate significant amount of heat on its own and should only be heated by the heater channel.

Since the response of the heater channel to the VOC was driven by self-heating of the channel, we named this type of response as *self-heating response*. However, the sensor channel only had a fixed dc bias which was not even large enough (only 100 mV dc) to invoke any significant heat transfer to the VOC molecules like the heater channel; hence any response by this channel was a result of the secondary heating of the sensor channel due to the higher sweeping bias of the heater channel and the associated response is called *secondary heating response*. The sensor channel is named so due to the fact that, in absence of VOCs, it transduces the change of temperature on the heater channel by acting as a temperature-dependent resistor. From Fig. 3(a), we see that the secondary heating response had a higher response magnitude compared to the self-heating response because of the absence of high electric field in the sensor channel.

Another important observation in Fig. 3(a) is the noticeable difference in bias voltage that triggered the onset of responses on both channels (V_th_), which was further investigated for other analytes. Figure 3(b) shows the linear trend between latent heat of evaporation (ΔH_vap_) and V_th_ for both channels for fifteen different VOCs covering widely varying classes of organic compounds, which establishes the universal nature of this correlation. The VOCs used as analytes are – N,N-dimethylformamide (DMF), hexane, methylbenzene (toluene), benzene, cyclohexane, cyclopentane, 1-propanol, 2-propanol (isopropanol), ethanol, methanol, trichloroethene, trichloromethane, ethoxyethane (diethyl ether), propanone (acetone) and ethanoic (acetic) acid glacial. The properties of these analytes are obtained from^45^ and are listed in Table 1. Although all three curves are linear, they have different slopes. The response from a TMH of comparable dimensions and fabricated monolithically on the same wafer is also presented as a reference. The curves for the self-heating response of MDC-MH and the TMH (reference device) are quite similar, which indicates that self-heating response is not strongly affected by the device geometry. However, the secondary heating response has a much higher slope (~0.32 V/mol.kJ) compared to the slope of the self-heating response (~0.14 V/mol.kJ). Since the separation between these channels at the tip is less than 1 μm (Fig. 1(b)) which indicates that at the tip region, the temperature difference between these two channels cannot be large enough to trigger this huge difference in response; hence the response is not purely a function of temperature only. The exact reason for such a deviation is still not clear, however we believe one possible explanation could be the difference in electric fields along the transport direction, which may have an effect on how analyte molecules interact with the polar surface of AlGaN. In order to verify the effect of surface interaction, we performed some experiments on the single channel TMH devices. Due to the absence of a second channel, TMH has fewer degrees of freedom and provides a simpler analog of the self-heating mechanism.

**Figure 4:**
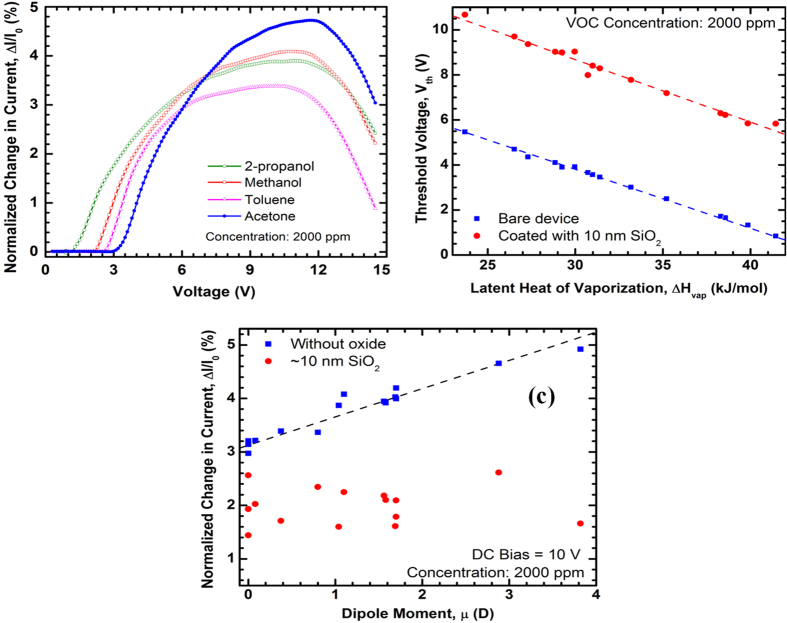
(**a**) Composite plots of normalized change in current (%) with change in voltage for 2-propanol, methanol, toluene and acetone at 2000 ppm molar concentration obtained using a reference TMH. A distinct voltage (V_th_) is exhibited for each analyte below which there is no detectable change in current. (**b**) Linear relation between the threshold voltage (V_th_) and latent heat of evaporation (ΔH_vap_) for different VOCs at 2000 ppm molar concentration. Note that the device coated with 10 nm SiO_2_ exhibits an upward shift in V_th_ by more than 4.5 V. (**c**) Correlation between normalized change in current (%) at 10 V dc bias and dipole moment (μ) for VOCs at 2000 ppm molar concentrations, taken from the same devices. For the oxide-coated device, there is no correlation between normalized change in current and molecular dipole moment, indicating a suppression of surface interaction promoted by the polar AlGaN surface that is only accessible on a bare device.

For this experiment, we took two identical TMHs with dimensions similar to the MDC-MH and fabricated on the same wafer. One of these devices was coated with 10 nm thick SiO_2_ (on the AlGaN surface) using plasma enhanced chemical vapor deposition (PECVD), while the other had a bare surface. First, we characterized the bare device to ensure that the device responded to the VOCs as expected (see Fig. 4(a)). Here we used four analytes (2-propanol, methanol, toluene and acetone), each with 2000 ppm of molar concentration, and observed the normalized change in current for each of them. The high concentration was used to maximize the signal-to-noise ratio of the response to determine whether a particular type of response is present or not. Just like the self-heating response in Fig. 3(a), the magnitude of the slope of ΔI/I_0_ starts to decrease after ~10 V. Next, we performed this experiment for all the 15 analytes for both of the TMHs (oxide coated and bare) and plot the V_th_ vs ΔH_vap_ curve for each of them in Fig. 4(b). Comparing these two linear trends, it is obvious that the bare-surface device responded to the VOCs at significantly lower threshold voltage level of ~4.5 V. This clearly indicates that the enhancement of device response due to the interaction of the VOCs with the polar AlGaN surface^48^. This interaction between polar molecules and polar surface becomes more apparent in Fig. 4(c), which presents the correlation between ΔI/I_0_ and molecular dipole moment (μ) of the VOCs at a constant dc bias (10 V) and the fixed analyte concentration (2000 ppm). We find that the device with bare AlGaN surface shows a linear correlation between the magnitude of ΔI/I_0_ and μ, however, the oxide coated device shows no correlation at all. While at a sufficiently high bias (at least several volts higher than V_th_) the response (ΔI/I_0_) is strongly affected by the interaction between polar molecules and the polar cantilever (AlGaN) surface^34^, in absence of a polar surface (like in the case of SiO_2_ deposited cantilever), ΔI/I_0_ becomes primarily dependent only on tip temperature and analyte concentration, and does not get affected by the molecular dipole moment, as observed in Fig. 4(c).

The observations in Fig. 4(b,c) clearly show that it is not the temperature alone that causes the microcantilever heaters to respond to the VOCs, which leads us to consider the effect of electric field at the surface as well. Since the tip region is narrower than the side arms, both of the MDC-MH channels have the highest resistance in this small part of the cantilever, accounting for the largest voltage drops across them. This builds up electric fields in the tip region that is larger in magnitude than anywhere else on the cantilever. Now, since self-heating mechanism involves a large sweeping voltage bias, as opposed to a fixed small dc bias associated with the secondary heating mechanism, the electric fields generated on both channels near the tip region would be significantly different. If a larger electric field aids in the surface interaction of analyte molecules (please refer to our earlier discussions), it should enhance the response of the device, which is why self-heating responses have lower V_th_ values than secondary heating response in Fig. 3(b).

While secondary heating response requires higher operating voltage for the heater channel, it brings in a significant advantage in terms of sensitivity. Here we introduce a parameter called *threshold voltage sensitivity* (S_v_) which indicates the change of V_th_ for a unit change in ΔH_vap_. This is defined as the magnitude of the slope of the fitted lines in Fig. 3(b). Higher S_v_ means the device has to be operated at larger bias voltages due to the larger window of V_th_, but that also offers better resolution as even small changes in V_th_ can be detected due to their wider separation and this helps in more accurate determination of unique VOCs. The general definition of S_v_ is,


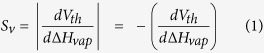


In Fig. 4(b), S_v_ = ~0.28 V/mol.kJ for both the bare and the oxide coated devices; so assuming that the surface temperature and electric field across the tip region for both these devices are the same, it is primarily the polar surface interaction that shifted the V_th_ for the oxide-coated device in upward direction without affecting the S_v_. However, in Fig. 3(b), where both channels have bare surfaces to allow surface interaction, we get S_v_ = 0.14 V/mol.kJ for self-heating response from the heater channel and S_v_ = 0.32 V/mol.kJ for secondary heating response from the sensor channel, the latter being about 120% higher than the former one. This observation leads us to the conclusion that the surface interaction promoted by the polar surface of AlGaN^48^ and the electric field have different effects. Even if the temperature of the tip is high enough to trigger a response in a self-heating setup and the surface of both channels are open to the VOCs, the secondary heating mode lacks the certain high electric field at the tip that allows manifestation of such changes. That is why an even higher bias across the heater channel is required to compensate for the absence of a strong electric field in the sensor channel which gives rise to different slope of V_th_ vs ΔH_vap_ curve as opposed to a mere upward shift as observed in Fig. 4(b). In order to account for the geometrical asymmetry of the channels, we also reversed the role of these channels (outer channel being the heater channel and inner channel being the sensor channel), but we still observed very similar numbers; S_v_ = 0.16 V/mol.kJ for self-heating response and S_v_ = 0.31 V/mol.kJ for secondary heating response. This eliminates the geometrical differences from being a significant factor for this large change in S_v_. It is obvious that the secondary heating mode, offered by the MDC-MH, significantly improves the accuracy and resolution of the sensor, at the expense of higher power dissipation in the heater channel. Any technique that increases the efficiency of the heater channel (i.e. achieve same temperature at a lower voltage) will unconditionally benefit the secondary heating response as the role of the heater channel is only to provide localized heating to the sensor channel at the tip; the voltage applied to the heater channel has no direct effect on the sensor channel. In Table S1 of the Supporting Information, we have listed the S_v_ for different devices.

**Figure 5:**
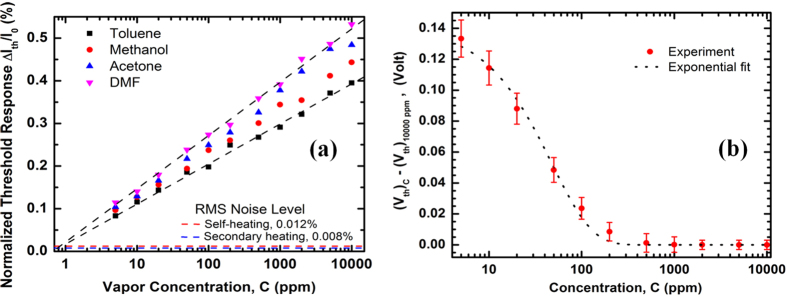
(**a**) Normalized threshold response, defined by the normalized change in current within a 20 mV voltage range around V_th_, shown as a function of concentration for four analytes. Threshold response varies linearly with logarithm of concentration, linear interpolations indicate a noise limited lower limit of detection below 1 ppm. The rms noise level of the entire detection set up is determined to be 0.012% for self-heating mode and 0.008% for secondary heating mode for <6 V bias. (**b**) Change in V_th_ is plotted as a function of concentration. At high concentration (>500 ppm) V_th_ is nearly constant, so that value is taken as the reference. At low concentration (5 ppm), V_th_ change is ~140 mV. The shift in V_th_ can be fitted reasonably well to a single exponential function, as shown in the figure. Since both self- and secondary heating modes indicated almost identical concentration-dependent deviation of V_th_, they are not shown separately.

In Fig. 5, we have presented the effect of analyte concentration on the response of MDC-MH with the goal of determining a limit of detection. We observed a maximum rms noise level of 0.012% for self-heating response and 0.008% for secondary heating response in ultra-high purity (UHP) N_2_ environment within the voltage range of 0–10 V. Therefore, V_th_ is arbitrarily defined by using a rms current magnitude change of at least 0.03% in presence of an analyte, which gives a signal to noise ratio of ≥ 2.5.

In order to quantify the magnitude of the device response at threshold voltage, we define the *normalized current response* at V_th_ (also known as the *normalized threshold response*) as,


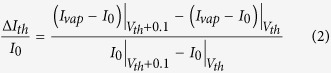


For any other voltage V > V_th_, normalized current response is defined as,


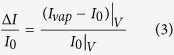


Here, the vertical bars followed by the voltage subscripts denote the voltages at which the corresponding currents (I_vap_–I_0_) and I_0_ are measured. I_vap_ and I_0_ are the currents measured with and without analyte vapor, respectively. V refers to the voltage across the heater channel only, for both types of responses. I_0_ can be obtained in real time using an identical reference cantilever or by calibrating the device before exposing to any VOC. It should be noted that, the normalized change in current for V > V_th_ (Equation (3)) is defined differently for the normalized threshold response in Equation (2), because the initial slope of the response curve around the V_th_ (given by ΔI_th_/I_0_) is a more reliable indicator for comparing the near-threshold response magnitude of different analytes than just ΔI/I_0_, at a point very close to the V_th_.

Comparing the response of the devices with different dimensions and wafer resistivity, it has been concluded that by making the tip region smaller in all three dimensions and reducing the voltage drop everywhere else, by using a wafer with higher conductivity, will generally help in achieving a better limit of detection (LOD) and noise limited resolution; although a predictive model to fine tune the geometry for obtaining the perfect balance of low and high resistance regions is yet to be developed. Using the present devices, we were able to reliably and repeatedly detect various VOCs selectively with a concentration down to 5 ppm as shown in Fig. 5(a). Here we used the expression of ΔI_th_/I_0_ defined in Equation (2) due to the higher degree of non-linearity in the device response around V_th_, as opposed to the general expression of ΔI/I_0_ (Equation (3)). The normalized threshold response (ΔI_th_/I_0_) for all four analytes asymptotically approached sub-ppm concentration with an overall rms noise level of 0.012%. It is expected for different analytes to give almost identical response at extreme dilution (sub-ppm in this case) as they approach the response of UHP N_2_ in that regime.

The relation between normalized threshold response (ΔI_th_/I_0_) and concentration can be expressed using a parameter that we call *threshold current sensitivity* (S_i_) which is defined as the difference in normalized threshold response, ΔI_th_/I_0_ for a tenfold change in concentration. This is mathematically defined as,


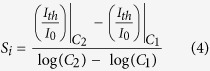


The larger the S_i_ is for a VOC, the easier it is to detect it and the limit of detection is likely to be lower for it for a given noise limit. From Fig. 5(a), S_i_ is 0.082%/decade for toluene and 0.14%/decade for N,N-dimethylformamide (DMF). In Table S2 of the Supporting Information, we have listed the S_i_ for some other analytes as well.

Figure 5(b) shows an important observation which is more apparent when noise is significantly suppressed using successive averaging as we have done here. As concentration goes down, V_th_ goes up slightly (only by ~130 mV as concentration goes down from 200 ppm to 5 ppm). Since the magnitude of response increases after bias voltage exceeds V_th_, it can be stated that higher bias makes the effect of VOCs more prominent. Since at lower concentration, there are fewer molecules to interact with the cantilever, a higher bias is required to compensate for it and make the effect measureable. However, above 200 ppm, V_th_ becomes constant regardless of concentration; which is evident in Fig. 5(b), where we used the response at 10,000 ppm as a reference. Since the shift of V_th_ at a particular concentration varies slightly for different analytes, we showed the range of ΔV_th_ for the analytes used in this study – toluene, methanol, acetone and DMF. For each concentration, the range of ΔV_th_ is shown in error bar and only the mid-range value is plotted. The small variation of V_th_ over the range of 5–10,000 ppm makes it very suitable for practical applications, such as indoor air quality monitoring system since most VOCs in household atmosphere have a concentration within that range^49^. The following empirical relation gives a good approximation of the ΔV_th_ with respect to concentration C, using V_th_ at 10,000 ppm as the reference −


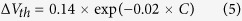


**Figure 6:**
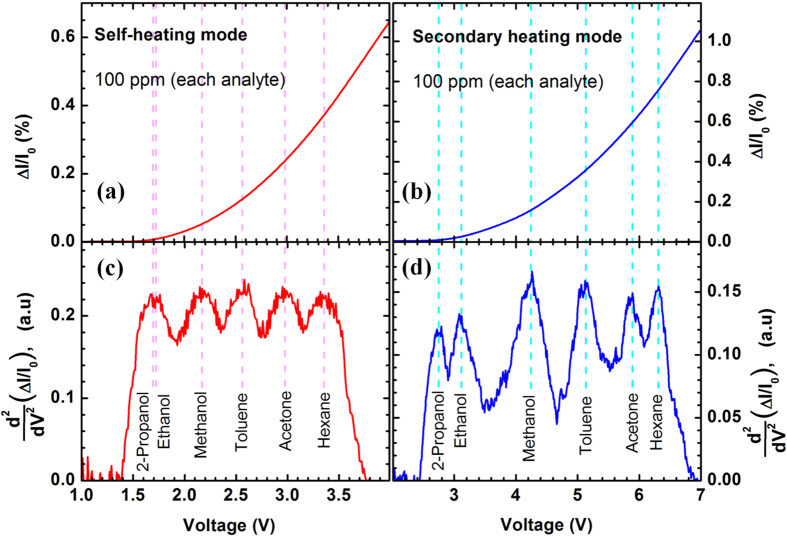
(**a**) Normalized change in current (%) obtained from a MDC-MH in self-heating mode, in presence of six analytes (100 ppm of each) –2-propanol, ethanol, methanol, toluene, acetone and hexane. (**b**) Normalized change in current (%) obtained from a MDC-MH in secondary heating mode for the same analytes. (**c**) The second order derivative of the response shown in (**a**) with respect to the applied bias (V) across the heater channel. Note that due to smaller threshold voltage sensitivity (S_v_), the peaks of 2-propanol and ethanol merged together in (**c**) and became indistinguishable. (**d**) The second order derivative of the response shown in (**b**) with respect to the applied bias (V) across the heater channel. Here, 2-propanol and ethanol peaks are clearly visible; the other peaks are well-separated from each other as well, allowing easier detection of the V_th_ peaks.

Mixture analysis is a very important aspect of analyte detection and often used as a benchmark metric for the detection technique. Figure 6 shows the response of MDC-MH device for a mixture of six analytes – ethanol, 2-propanol, methanol, toluene, acetone and hexane; each with 100 ppm concentration. In Fig. 6(a), we see the normalized response from the heater channel in self-heating mode where the response is observed to rise non-linearly after the bias exceeded 1.7 V. However, this plot exhibits no clear indication of multiple VOCs being present in the system. Similarly, Fig. 6(b) shows the secondary heating response from the sensor channel of the device during the same measurement, where the response starts to rise from 2.75 V without showing any noticeable sign of multiple VOCs. We went ahead to further examine the first and second order derivatives of (a) and (b) with respect to the applied bias across the heater channel, to look for possible VOC signature responses. We did not find any noticeable peaks in the first order derivative (please refer to Figure S2 of the Supporting Information); interestingly however, the second order derivatives clearly showed multiple peak positions. Figure 6(c,d) present the second derivatives of Fig. 6(a,b) respectively. In Fig. 6(c), we can clearly distinguish five peaks; and after comparing the peak locations with the V_th_ for the same set of analytes in Fig. 3(b) (self-heating response), we observe that these peak locations closely overlap with those V_th_ values. Since out of the six analytes in the system, two (2-propanol and ethanol) had very close V_th_s, their individual peaks were indistinguishable in Fig. 6(c). The other four analytes had well-separated V_th_s and their peaks could be identified fairly easily. However, in Fig. 6(d), which shows the second order derivative for the response obtained using secondary heating mode, we can clearly identify six individual and more sparsely located peaks, whose locations match closely with the V_th_s for the corresponding analytes shown in Fig. 3(b). The better detectability of the peaks for the secondary heating mode can be attributed to the higher S_v_ of this mode, which is associated with a larger spacing of the V_th_s for a set of analytes. A direct comparison between the two sensing modes utilizing a mixture of several VOCs clearly highlights the advantages of the secondary heating mode in distinguishing analytes, especially those with closely matched physical/chemical properties.

We also performed Gaussian fitting of the peaks, which revealed a generic correlation between full width at half maximum (FWHM) of the peaks and concentration of the analytes. The average FWHM of the peaks in secondary heating mode for all the analytes stayed within 0.35–0.5 V for 10 ppm, which decreased to the range 0.25–0.35 V for 100 ppm, and finally to 0.1–0.25 V for all concentrations above 500 ppm. A more complete list of values of FWHM at different concentrations and from both self-heating and secondary heating modes is given in Table S4 of the Supporting Information, we also listed the ΔH_vap_ and V_th_ from different devices in Table S3. It has been observed that the FWHM of the peaks are smaller for secondary heating mode, which makes them easier to distinguish from adjacent peaks. Also, the V_th_ (i.e. location of the peaks) for both modes matched very closely to the ones shown in Fig. 3(b), although those measurements were performed using single analytes. In order to further verify the stability of the V_th_ values, we repeated the multi-VOC sensing measurements with 2, 3 and 4 different analytes, and observed virtually no shift in V_th_ for any combination of analytes (please refer to Table S5 of the Supporting Information for further details).

While the MDC-MH offers many desirable features of a VOC sensor, it does not provide adequate information about the concentration of the vapor being detected, which is equally important for practical applications. While at low concentrations, FWHM of the peaks may be used to approximately estimate the concentration (see above discussion), the resolution in terms of detected concentration is insufficient for robust practical applications. In order to address this limitation, we have designed and studied another type of dual channel microcantilever heater with the capability to offer a much better estimate of the VOC concentration. Their construction is very similar to the MDC-MHs, except that the tip of the cantilever is split (and hence the name being *split tip dual channel microcantilever heater* or SDC-MH) which physically separates the two channels of the cantilever along more than 50% of its length. The gap between these two channels at the tip edge is less than 1 μm lithographically, but the bending of the cantilever makes this gap more than 1 μm. Additionally bending of the cantilever arms tends to bring the outer channel partially above the inner channel (refer to SEM images in Fig. 1) which can allow convection current from the heater channel to come in contact to the sensor channel and serve as an additional mode of heat transfer. Just like the MDC-MH, we used the outer and inner channels of the SDC-MH as the sensor and heater channels respectively. While the self-heating response of SDC-MH is almost identical to MDC-MH, the secondary heating mode for SDC-MH gives relatively weaker response compared to the MDC-MH due to the absence of a direct physical connection between the tips, which significantly lowers the heat transfer efficiency.

**Figure 7:**
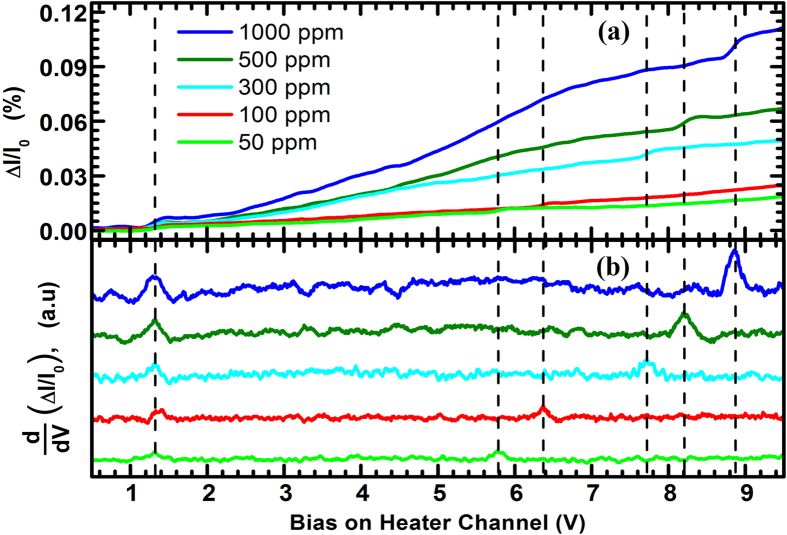
(**a**) Normalized change in current (%) for 50, 100, 300, 500 and 1000 ppm of 2-propanol, as measured using a split tip dual channel microcantilever heater (SDC-MH) in secondary heating mode (fixed 100 mV dc bias across the sensor channel). (**b**) The first order derivative of the response curves shown in (**a**), with respect to heater channel bias. Each curve shows two peaks for the same analyte, the first one (within 1.2–1.4 V) indicates the onset of the sensing behavior on the heater channel, while the second peak, at much higher bias, is due to the sensing effects exhibited by the sensor channel.

In Fig. 7(a), the normalized change in current (ΔI/I_0_) is presented as a function of bias voltage across the heater channel for five different concentrations of 2-propanol (50, 100, 300, 500 and 1000 ppm). We observe that even for a single analyte, each curve exhibits small but abrupt changes in two different voltages, which we did not observe for the MDC-MHs . The first derivatives of these response curves, shown in Fig. 7(b), reveal two clear peaks on each curve. The first peak (designated *the lower order V*_*th*_ or *V*_*th,low*_), located at about 1.3–1.4 V, remains almost constant regardless of the concentration. Measuring the self-heating response of the heater channel in presence of the same analyte showed that V_th_ for 2-propanol was 1.1 V which was slightly lower than the V_th,low_ (1.3–1.4 V) observed in Fig. 7(b). While it is obvious that V_th,low_ is directly related to the V_th_ of the self-heating event, a similar peak (analogous to V_th,low_) was not observed in the secondary heating mode of the MDC-MH. Since the heat transfer mechanisms are very different for MDC-MH and SDC-MH, and the relative distance between the two channels of the SDC-MH also varies due to the uneven thermal expansion of the split tip, the exact origin of this variation in response requires further study.

The second peak that occurs at a higher voltage on each curve of Fig. 7(b) (designated *the higher order V*_*th*_ or *V*_*th,high*_) is analogous to the V_th_ of the secondary heating response of the MDC-MH, as this is a result of the sensor channel being heated up by the heater channel. However, V_th,high_ shifts to higher voltages as the concentration of the analyte goes up. The presence of the analyte at a higher concentration results in a more efficient heat loss from heater channel, which causes its temperature to be lower than what it would be with a less concentrated analyte vapor. The lower temperature of the heater channel also lowers its thermal expansion and causes it to stay further away from the sensor channel. The lower temperature of the heater channel coupled with the increased distance between the two channels in presence of a more concentrated analyte reduces the heating rate of the sensor channel significantly, which is reflected in the increasing magnitude of the V_th,high_. As seen in Fig. 7(b), while the concentration of 2-propanol changed from 50 ppm to 1000 ppm (20 times), V_th,high_ shifted by about 3.1 V.

**Figure 8:**
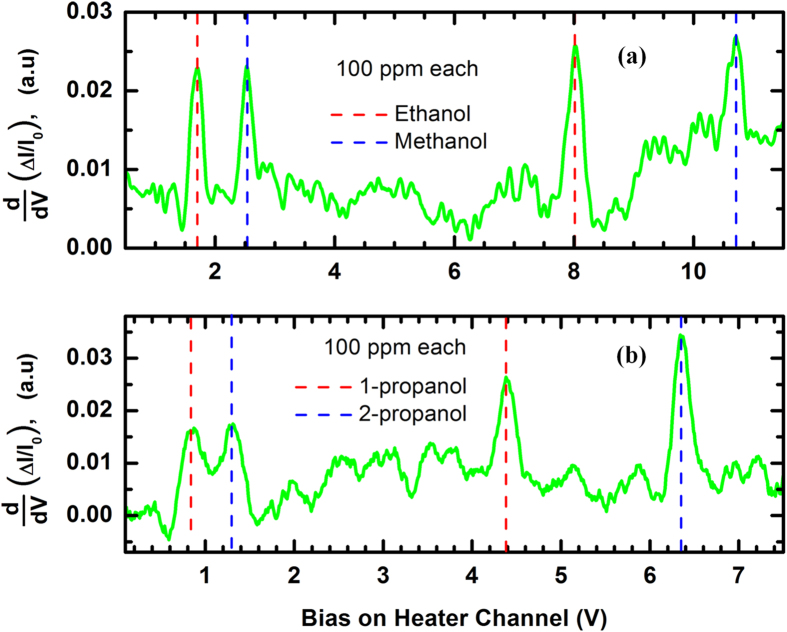
First order derivative of the normalized change in current (%) obtained from the SDC-MH for a mixture containing 100 ppm (each) of (**a**) ethanol and methanol; (**b**) 1-propanol and 2-propanol. Each analyte exhibits a lower voltage peak corresponding to self-heating sensing, and a higher voltage peak indicative of secondary heating induced sensing.

Like MDC-MH, the SDC-MH was also found to be capable of simultaneous detection of multiple VOCs. Figure 8(a) shows the results of sensing experiments carried out with a mixture of 100 ppm each of methanol and ethanol, while Fig. 8(b) shows the responses for a mixture of 1-propanol and 2-propanol (each of 100 ppm concentration). It should be noted that 1-propanol and 2-propanol are isomers with very similar properties, still they can be detected due to the small difference in their ΔH_vap_s. It can be seen that in all four cases, the V_th,low_ is 150–200 mV higher than the V_th_ detected by the self-heating mode.

The lower order V_th_s (V_th,low_) of the secondary heating response are detected directly from the self-heating response of the heater channel (SDC-MH) or from the self- or secondary heating response of a similar MDC-MH. These peaks are then identified in the first order derivative of the secondary-heating response (d/dV of ΔI/I_0_) by searching for the largest peaks around the estimated locations of the V_th,low_s. Then we compare this peak height with the rms value of the random fluctuation. If the V_th,low_ peak is not larger than the rms fluctuation by a certain factor (1.25 in the current work, determined after many test runs at various concentrations), we increase the averaging window to lower the noise until the V_th,low_ peak height is larger than the rms noise level by at least 1.25 times. The averaging window is allowed to grow by a maximum of 200 cycles instead of the regular 50 cycles averaging scheme used in other measurements.

**Figure 9:**
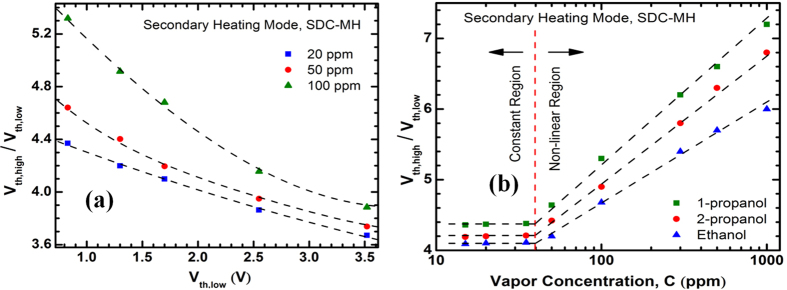
(**a**) Ratio of the higher and lower order V_th_ values (V_th,high_/V_th,low_) as a function of V_th,low_ for different vapor concentration. At low concentrations (10–40 ppm), the relation is almost linear. Above 40 ppm, the relation becomes significantly non-linear; especially for low-V_th_ analytes. (**b**) Ratio of the higher and lower order V_th_ values (V_th, high_/V_th, low_) plotted against vapor concentration, showing how this ratio changes for each analyte. Notably, above 40 ppm, the ratio for each analyte is a nice logarithmic function of concentration, primarily depending on the equivalent thermal conductivity of the vapor-air mixture.

In order to find out the relation between V_th,high_ and concentration, we have defined a parameter R_v_, a unit less number, which is the ratio of the higher and lower order V_th_ values (V_th,high_/V_th,low_). In Fig. 9(a), we plot R_v_ vs. V_th,low_ for 1-propanol, 2-propanol, ethanol, methanol and trichloroethene with concentrations of 20, 50 and 100 ppm. At 100 ppm, there exists a highly non-linear trend which gradually tends to become linear as concentration goes down, as seen at 20 ppm where it becomes almost linear. From our previous discussion, V_th,high_ becomes concentration-dependent due to the combined effects of at least two factors – (1) different amounts of heat loss due to the concentration variation, and (2) different amounts of thermal expansion (i.e. gap between the channels) due to the result of (1). The outcome of these two factors is a non-linear dependence of R_v_ on concentration. However, at lower concentrations, the change in thermal characteristics of the heater channel is much smaller due to the fewer number of molecules interacting with it. This reduces the impact of the two factors that resulted in the concentration-dependence of R_v_. Therefore, the dependence of V_th,high_ on concentration reduces greatly, and R_v_ exhibits an almost linear relation with V_th,low_.

The aforementioned assertion is further illustrated in Fig. 9(b), where R_v_ for three anaytes (1-propanol, 2-propanol and ethanol) are shown for concentrations ranging from 20 ppm to 1000 ppm. We see that above ~40 ppm, R_v_ is a logarithmic function of concentration, C. However, below 40 ppm, R_v_ becomes essentially constant for different concentrations. This trend is in good agreement with our discussion above, and can be used to determine the concentration of an analyte in the range of 40–2000 ppm. It should also be noted that, due to the weaker coupling between the channels in SDC-MH in comparison with MDC-MH, the secondary heating mode of SDC-MH has an inferior lower limit of detection (~10 ppm). This can be improved by reducing the gap between the two channels.

Since R_v_ (i.e. V_th,high_) is a strong function of concentration, there is a possibility that if there are several VOCs with different concentrations in a mixture, V_th,high_ of one analyte may become smaller than V_th,low_ of another and cause erroneous interpretation of the observation. To avoid such a scenario, both MDC-MH and SDC-MH should be used in an array to combine their unique features and obtain better detection results by following an improved algorithm discussed below. The MDC-MH, in its secondary heating mode, will identify the analytes with high resolution and provide an initial estimate of concentration based on the FWHM of the individual peaks. Then from the secondary heating response of the SDC-MH, V_th,low_s can be identified using the V_th_s given by the MDC-MH, which should be very close. Finally, based on the initial estimate of the concentrations given by the MDC-MH, V_th,high_s of the SDC-MH can be determined to pair up with their respective V_th,low_ values. The calculation of R_v_ can then lead to a more accurate determination of concentration of each analyte.

All the results presented in this work were produced in dry UHP nitrogen background, which ensured a uniform and regulated background for the study. However, in real life applications, the device would be exposed to regular air containing oxygen, humidity, other gases etc. We have observed no interference from water vapor (low or high concentration), which was also presented in our previous report^34^. The presence of oxygen also did not create any interference, which was expected as the device operated below the auto-ignition temperature of the analytes and hence ruled out the possibility of combustion to take place. Similarly CO_2_ or other trace gases in the air did not modify the response in the test cases; and the interference from other background VOCs can also be dealt with using the selective detection schemes presented earlier in the work.

The safe operating limit of these devices was determined to be 15 V; within this limit these devices could be operated for a very long time. Our experiments were performed for a period of over a year, without the cantilever exhibiting any noticeable degradation or drift in performance. Since the primary sensor output is of normalized differential nature (ΔI/I_0_), it is expected that even small changes in the device behavior over a longer period of time would not significantly affect the calibration of these devices.

## Conclusions

In conclusion, we have demonstrated highly sensitive and selective detection of a wide range of volatile organic compounds (VOCs), individually and in mixture, utilizing specially designed AlGaN/GaN heterostructure based dual-channel microcantilever heaters. Utilizing separate heater and sensor channels these microcantilevers heaters demonstrated much superior sensing capability compared to the single-channel ones, and were able to perform detection over a wide concentration range of 5–10,000 ppm with detection limit in the sub-ppm range, as well as uniquely identify the components in a mixture of 6 analytes. Analyte detection was performed without any functionalization of the cantilevers, solely utilizing their unique channel geometry and polar nature of the AlGaN surface, with the analytes differentiated based on their established physical properties of latent heat of evaporation and dipole moment. An algorithm for analysis of complex mixtures, including identification of the analytes present and estimation of their concentrations, has been presented, which can be easily implemented in practice utilizing an array of these microcantilevers simultaneously operating in different sensing modalities. The novel detection results presented open up exciting possibilities for developing integrated VOC detection chips for miniaturized handheld sensor systems that can find widespread usage in industrial, medical and home-based monitoring applications.

## Materials and Methods

The microcantilevers used in this work were fabricated using AlGaN/GaN heterostructure epitaxial layers grown on a 700 μm thick (111) Si substrate. The microcantilevers were designed to have tapered arms (shown in Fig. 1) to maximize the temperature rise at their tip under an applied bias. Both the MDC-MH and SDC-MH devices had a base width of 30 μm, tip width of 4–5 μm and channel separation of about 1 μm at the tip. Details of the wafer structure and the fabrication process are given in Section VI of the Supporting Information.

The experimental setup used for sensing volatile organic compounds involved a homemade test chamber fitted with vapor inlet and an outlet. One mass flow controller (MFC) was used to control the composition of the VOC vapor produced by flowing ultra-high purity (UHP) N_2_ carrier gas through a bubbler maintained at a constant temperature of 30 °C. The bubbler temperature was maintained using temperature-controlled hot plate (Thermo Scientific HP131535) to compensate for the heat loss due to evaporation, to make sure that the generated vapor stayed nearly at room temperature. A solenoid valve was used to control the duty cycle of the vapor flow in to a dilution chamber where UHP N_2_ from another MFC was mixed with the incoming vapor flow from the bubbler. The duty cycle of the bubbler outlet and the ratio of the vapor/N_2_ flow rates were adjusted to obtain the desired concentration of the vapor. The concentrations of the analytes were estimated using van der Waal’s equation as the dilute vapor/N_2_ mixtures would behave like pure N_2_. Following widely accepted practice, we assumed a saturated vapor of the organic compound to prevail inside the bubbler, which was subsequently diluted by the dry N_2_, where the concentration was adjusted by varying the ratio of the vapor/N_2_ flow rates and by tuning the duty cycle of the mixing assembly (for further dilution). For further verification, we applied this technique for generating water vapor as well, where the estimated level of humidity closely matched with the value measured by a psychrometer. For experiments with multiple VOCs, each VOC was put in its own MFC/bubbler assembly and the vapors from such channels were mixed in the dilution chamber with N_2_ to produce the mixed vapor. After every experiment, the test chamber was flushed with UHP N_2_ to allow for recovery of the sensor device. The electrical and sensing characterizations were performed using Agilent B2902 and Keithley 2612A source measure units (SMU). For all sensing experiments, a continuous voltage sweep with a resolution of either 100 mV (single analyte detection) or 10 mV (simultaneous multiple VOC detection) was used. The sampling rate that was used in all measurements was 1 kHz, which took 0.2 s to complete a triangular sweep between 0 and 10 V with 100 mV resolution. We varied the sampling rate between 500 Hz and 3 kHz and did not notice any significant change in device responses, except for the magnitude of the signals (i.e ΔI/I_0_) which reduced by no more than 5% as sampling rate increased from 1 kHz to 3 kHz. The sampling rate of 1 kHz allowed the sensor to take 50 readings at 100 mV resolution within 10 s, which enables the system to gather sufficient number of samples for averaging and noise reduction while being fast enough for practical application.

Three devices of each type were tested for each experiment and they all exhibited similar trends, while the values differed slightly which could be attributed to process variations during the fabrication of the chips. The behavior of the TMH was very similar to the ones presented in our earlier report^34^, which was fabricated on a different wafer and the process parameters were also different. The result of each experiment was obtained by averaging 50 consecutive sweep cycles (triangular waveforms used in sweeping), which allowed 100 readings of each bias point to be averaged. The measurements were repeated at least 5 different times over a period of one year for re-confirmation, though no detectable drift was observed at any time. The most representative results (from at least 5 instances of readings) were used in this article; instead of averaging all of those different readings, which would lower the noise level further and overestimate the detection limit of the sensor. For secondary-heating response of SDC-MH, we used a variable length averaging window, which spanned between 50 and 200 triangular sweep cycles.

## Additional Information

**How to cite this article**: Jahangir, I. and Koley, G. Dual-channel microcantilever heaters for volatile organic compound detection and mixture analysis. *Sci. Rep.* **6**, 28735; doi: 10.1038/srep28735 (2016).

## Supplementary Material

Supplementary Information

## Figures and Tables

**Table 1:** Molar latent heat of evaporation (ΔH_vap_) and dipole moment (μ) of different analytes.

Analyte	ΔH_vap_ (kJ/mol)	μ (D)	Analyte	ΔH_vap_ (kJ/mol)	μ (D)
Diethyl Ether	26.52	1.1	2-Propanol	39.85	1.58
Acetone	30.99	2.88	Benzene	30.72	0
Hexane	28.85	0.08	Cyclohexane	29.97	0
DMF	38.30	3.82	Cyclopentane	27.30	0
Toluene	33.18	0.375	1-Propanol	41.44	1.56
Trichloroethene	31.40	0.8	Trichloromethane	29.24	1.04
Methanol	35.21	1.7	Acetic acid	23.70	1.7
Ethanol	38.56	1.69			

Values are taken from^47^.
