# Supplementary material for: Dual-channel microcantilever heaters for volatile organic compound detection and mixture analysis
Source: Sci Rep. 2016 Jul 6;6:28735. doi: 10.1038/srep28735 (PMC4933970; doi:10.1038/srep28735)
Supplement: Supplementary Information [file srep28735-s1.pdf]

Supporting Information

Dual-channel microcantilever heaters for volatile organic compound detection and mixture analysis

Ifat Jahangir, and Goutam Koley*

(I) Electrical characterization of the sensor channels

The current-voltage (I-V) characteristics of the sensor channels of the MDC-MH and SDC-MH are shown in **Figure S1**(a) where strong non-linearity is observed. The currents are observed to decrease after ~5 V, which also happened for the heater channel. **Figure S1**(b) shows how power and resistance changes for the sensor channel of either device as the voltage bias varies. Here we see the resistance to increase by almost 275% for SDC-MH and 380% for MDC-MH as the bias changes from 0 to 14 V, with maximum power dissipation below 3 mW.

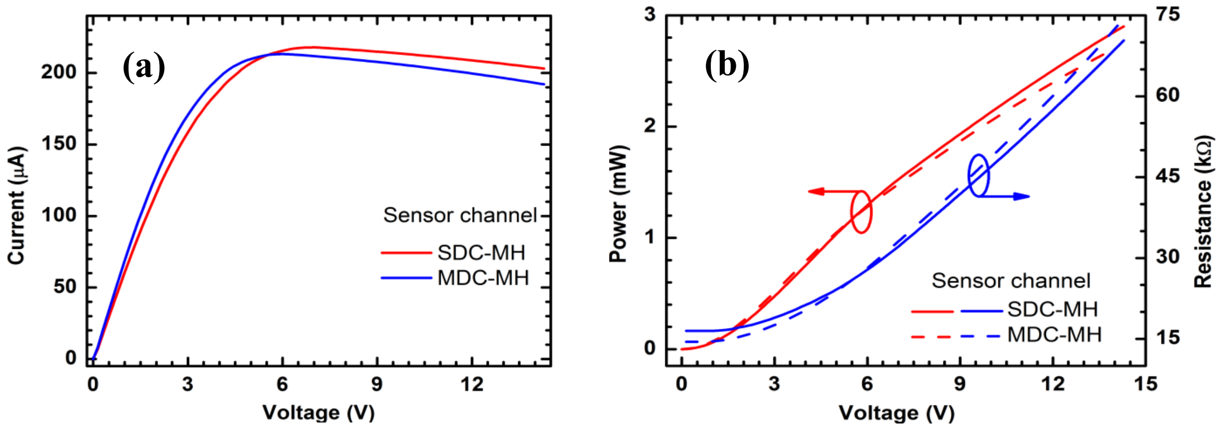

Figure S1. (a) I-V characteristics of the sensor channels of monolithic and split tip dual channel microcantilever heaters (MDC/SDC-MH). (b) Variation in consumed power and channel resistance with bias voltage obtained from the I-V characteristics in (a).

(II) Sensitivity of MDC-MH and SDC-MH

We have defined two types of sensitivity parameters in this work, the first one is *threshold voltage sensitivity* (S_v) and the second one is *threshold current sensitivity* (S_i). Their general definitions are (from **Equation** (1) and **Equation** (4) of the main article) –

$$S_v = \left| \frac{dV_{th}}{d\Delta H_{vap}} \right| = -\left(\frac{dV_{th}}{d\Delta H_{vap}} \right) \quad \text{and} \quad S_i = \frac{\left(\frac{I_{th}}{I_0}\right)\Big|_{C_2} - \left(\frac{I_{th}}{I_0}\right)\Big|_{C_1}}{\log(C_2) - \log(C_1)}$$

While S_v is an intrinsic property of the sensor, S_i is a property that is both device-specific and analyte-specific. In **Table S1**, we have listed the S_v for various devices used in this work. For SDC-MH, the definition of S_v is not valid in secondary-heating mode due to the non-linear nature of the response. Also we have shown the S_v for two different prototypes for each type of dual channel microcantilever heater to demonstrate the consistency in the observations. **Table S2** shows the S_i for several analytes from different devices in both self-heating and secondary heating mode.

Table S1: Threshold voltage sensitivity (S_v) of different microcantilever heaters

Device/Channel	Self-heating mode S_v (V/mol.kJ)	Secondary heating mode S_v (V/mol.kJ)
TMH (bare)	0.281	Mode not available
TMH (oxide coated)	0.279	Mode not available
MDC-MH(device 1)/Heater	0.142	0.311
MDC-MH(device 1)/Sensor	0.158	0.319
MDC-MH(device 2)/Heater	0.175	0.343
MDC-MH(device 2)/Sensor	0.171	0.340
SDC-MH(device 1)/Heater	0.163	Definition not valid
SDC-MH(device 1)/Sensor	0.159	Definition not valid
SDC-MH(device 2)/Heater	0.154	Definition not valid
SDC-MH(device 2)/Sensor	0.148	Definition not valid

Table S2: Threshold current sensitivity (S_i) of different analytes

Analyte	S_i (%/decade) MDC-MH (Self-heating)	S_i (%/decade) MDC-MH (Sec. heating)	S_i (%/decade) SDC-MH (Self-heating)	S_i (%/decade) TMH (Self-heating)
Diethyl Ether	0.095	0.097	0.096	0.092
Acetone	0.119	0.122	0.120	0.116
Hexane	0.043	0.045	0.043	0.041
Dimethylformamide	0.144	0.146	0.144	0.138
Toluene	0.082	0.083	0.083	0.079
Trichloroethylene	0.091	0.092	0.092	0.088
Methanol	0.103	0.105	0.104	0.100
Ethanol	0.102	0.104	0.102	0.101
2-Propanol	0.099	0.101	0.099	0.096
1-Propanol	0.099	0.100	0.100	0.095

(III) Comparison between the first and the second derivatives of $\Delta I/I_0$

When multiple VOCs are sensed by the MDC-MH, TMH and SDC-MH (only self-heating mode), the response itself does not give any clear indication of more than one analytes being present in the environment. Therefore, we take the first and second derivatives with respect to heater channel bias to detect small abrupt changes on the responses. In **Figure S2**, subplots (a), (b), (e) and (f) are reproduced from **Figure 6** of the main article, while (c) and (d) represent the first derivatives of (a) and (b). It is obvious that the first derivatives do not provide any distinct indication of multiple VOCs being detected; unlike the second derivatives, which show sharp peaks at the V_{th} of each analyte.

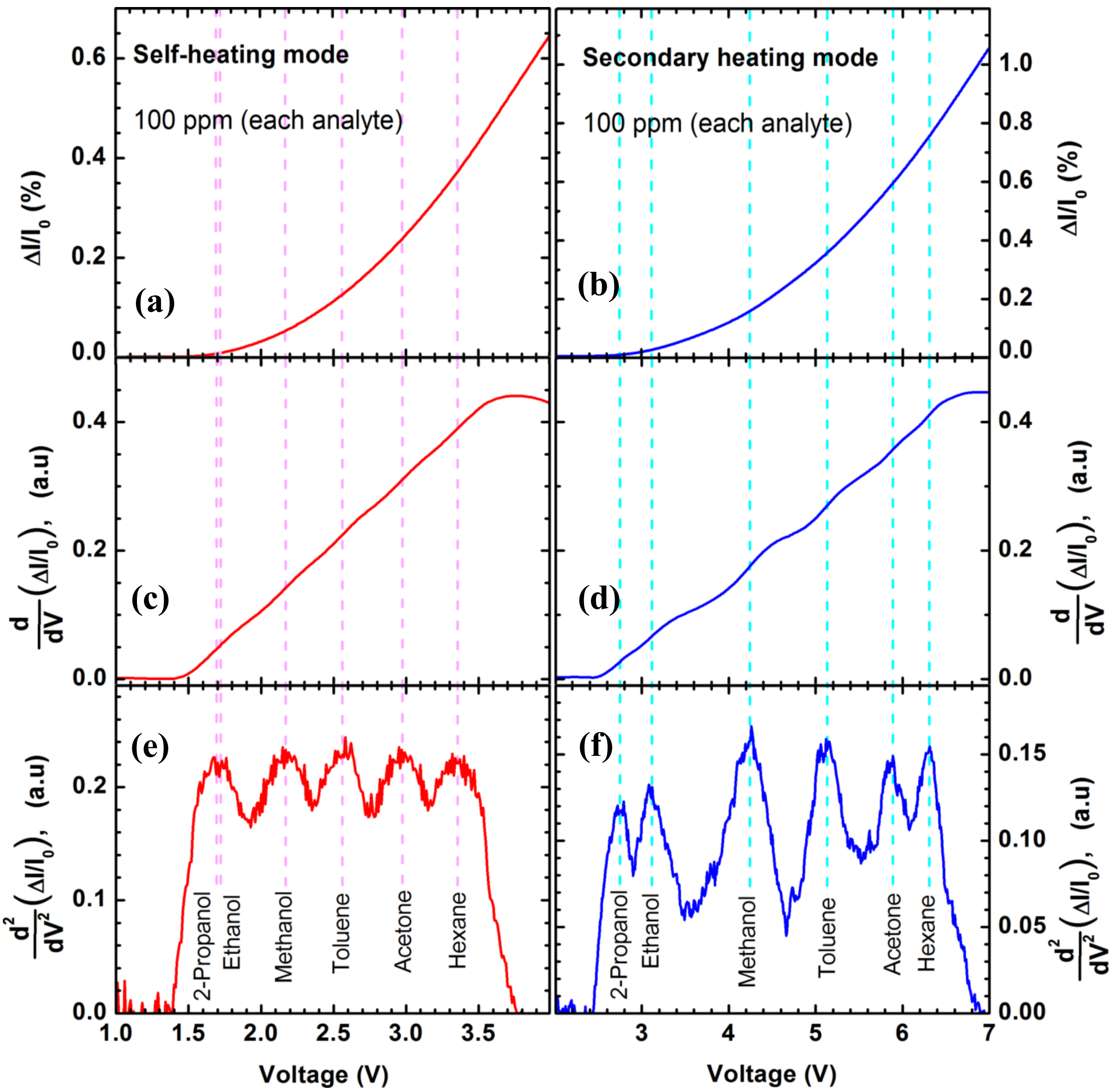

Figure S2: (a), (b) Normalized change in current (%) obtained from a MDC-MH in (a) self-heating and (b) secondary heating modes, reproduced from **Figure 6**(a), (b) of the main article. (c) First derivative (with respect to the bias applied to the heater channel) of the response shown in (a). (d) First derivative (with respect to the bias applied to the heater channel) of the response shown in (b). (c) and (d) are the second derivatives of (a) and (b) respectively, and reproduced from **Figure 6**(c), (d) of the main article.

(IV) Threshold Voltage and FWHM for different analytes and concentrations

We have observed different V_{th} for the same analyte using different devices due to the differences in S_v. We are showing the V_{th} for both modes of the MDC-MH and the self-heating mode for the SDC-MH and the TMH in **Table S3**.

In **Table S4**, we have listed the FWHM of the peaks, observed in the second derivative of the responses, for the MDC-MH, SDC-MH and TMH devices in presence of various analytes at different concentrations. The experiments were performed using a single analyte or a mixture of analytes.

Table S3: Threshold voltage (V_{th}) and ΔH_{vap} of different analytes

Analyte (1000 ppm)	ΔH_{vap} (kJ/mol) ‡	V_{th} (V) Sec. heating, MDC-MH	V_{th} (V) Self-heating, MDC-MH	V_{th} (V) Self-heating, SDC-MH	V_{th} (V) Self-heating, TMH
Diethyl Ether	26.52	7.11	3.57	3.74	3.93
Acetone	30.99	5.87	2.95	2.98	3.02
Hexane	28.85	6.31	3.34	3.40	3.45
DMF	38.30	3.47	1.93	1.74	1.54
Toluene	33.18	5.11	2.57	2.58	2.58
Trichloroethene	31.40	5.65	2.71	2.83	2.94
Methanol	35.21	4.22	2.14	2.15	2.17
Ethanol	38.56	3.10	1.67	1.59	1.49
2-Propanol	39.85	2.72	1.66	1.45	1.23
Benzene	30.72	5.81	2.85	2.98	3.10
Cyclohexane	29.97	6.04	3.14	3.23	3.30
Cyclopentane	27.30	6.82	3.37	3.51	3.65
1-Propanol	41.44	2.43	1.44	1.14	0.85
Trichloromethane	29.24	6.45	3.11	3.20	3.29
Acetic acid	23.70	7.95	3.90	4.21	4.54

‡ Values taken from [1].

Table S4: FWHM of the detection peaks for different analytes at different concentration

Number of analytes* in the mixture	Concentration (ppm)	FWHM (mV) MDC-MH (Sec. heating)	FWHM MDC-MH (Self-heating)	FWHM SDC-MH (Self-heating)	FWHM TMH (Self-heating)
1	5	0.51	0.53	0.55	0.54
1	10	0.43	0.47	0.48	0.50
1	50	0.35	0.38	0.40	0.42
1	100	0.29	0.33	0.34	0.34
1	200	0.25	0.27	0.28	0.30
1	500	0.21	0.23	0.23	0.24
1	1000	0.14	0.17	0.16	0.20
5	5	0.55	0.53	0.58	0.61
5	10	0.48	0.51	0.51	0.53
5	50	0.39	0.41	0.43	0.43
5	100	0.31	0.34	0.32	0.36
5	200	0.27	0.29	0.29	0.32
5	500	0.20	0.24	0.22	0.25
5	1000	0.13	0.17	0.17	0.20

* Same sets of analytes were used in these experiments. FWHMs for all similar experiments were averaged.

(V) Shift in V_{th} in presence of multiple analytes

It has been observed that for self- and secondary heating modes, the V_{th} does not shift noticeably regardless of the number of VOCs present in the system. This makes this technology an ideal solution for analyzing components of a mixture of unknown VOCs of various concentrations. In **Table S5**, we present the V_{th} for 100 ppm of ethanol obtained from various devices, with or without other analytes being present and detected simultaneously.

Table S5: Threshold voltage of ethanol in presence of other analytes

Device/mode	Other analytes (concentration in ppm, in parentheses)	V_{th} of ethanol (V)
MDC-MH Self- heating	None	1.68
	methanol (200), toluene (100), hexane (200)	1.67
	acetone (100), toluene (200), DMF (100), hexane (200)	1.68
	1-propanol (100), hexane (100), 2-propanol (200)	1.67
MDC-MH Sec. heating	None	3.11
	methanol (200), toluene (100), hexane (200)	3.11
	acetone (100), toluene (200), DMF (100), hexane (200)	3.10
	1-propanol (100), hexane (100), 2-propanol (200)	3.11
SDC-MH Self-heating	None	1.60
	methanol (200), toluene (100), hexane (200)	1.60
	acetone (100), toluene (200), DMF (100), hexane (200)	1.61
	1-propanol (100), hexane (100), 2-propanol (200)	1.61
TMH Self- heating	None	1.50
	methanol (200), toluene (100), hexane (200)	1.49
	acetone (100), toluene (200), DMF (100), hexane (200)	1.50
	1-propanol (100), hexane (100), 2-propanol (200)	1.49

(VI) Fabrication details for the microcantilever heaters:

We used deep anisotropic silicon etch technique to achieve suspended cantilever structure, where silicon was the sacrificial layer. All the fabrication steps were carried out in the Institute of Electronics and Nanotechnology (IEN) facility at Georgia Institute of Technology, Atlanta, GA. We started our process with 1.8 cm by 1.8 cm diced pieces of 6 inch AlGaN/GaN HEMT Epi wafer grown on Silicon (111) substrate, purchased from NTT Advanced Technology Corporation, Japan. The wafer had 2 nm iGaN and 15 nm $Al_{0.25}Ga_{0.75}N$ on 1 μm iGaN, with 300 nm buffer layer separating the GaN layer from 750 μm Si substrate.

At first, using a SiO_2 hard mask deposited by plasma enhanced chemical vapor deposition (PECVD) technique, we used an inductively coupled plasma (ICP) etcher with Cl_2/BCl_3 to isolate the cantilever mesa, leaving AlGaN layer intact only on the cantilever. Then ICP etching of GaN was performed to make an outline of the cantilever while AlGaN mesa was protected by 1 μm PECVD SiO_2 which was later etched away by HF wet etching. Then 20/100/45/55 nm Ti/Al/Ti/Au metal stack was deposited using e-beam metal evaporator on the cantilevers at the bases, followed by annealing at 800˚C in presence of N_2 for about 60 s to make good ohmic contact. Then the metal contact pads were formed using another stage of lithography and deposition of Ti/Au contacts.

After that the Si at the bottom of the pocket was etched from the backside of the sample. The back pocket outline was defined by a patterned SiO_2 mask layer aligned with the top pocket and then "Bosch process" was used to etch Si using ICP etcher.[2]-[4] Finally the square chips were attached to a dual-in-line (DIP) chip carrier package and all contact pads wire bonded

with Au wire. The length of each cantilever arm from base to tip was 130 μm, width was 30 μm at the base. The arms were separated by a 100 μm gap at the base. The tip width was about 4-5 μm, where each channel had a width of about 1.5-2 μm at the tip. The thickness of the cantilever was ~700 nm.

References:

[1] Lide, D. R. CRC Handbook of Chemistry and Physics, 90th edition, *CRC Press* (2010).

[2] Talukdar, A., Qazi, M. & Koley, G. High frequency dynamic bending response of piezoresistive GaN microcantilevers. *Appl. Phys. Lett.* **101** (25), 252102 (2012).

[3] Davies, S. et al. Fabrication of GaN cantilevers on silicon substrates for microelectromechanical devices. *Appl. Phy. Lett.* **84** (14), 2566-2568 (2004).

[4] Jahangir, I., Quddus, E. B. & Koley, G. Unique detection of organic vapors below their auto-ignition temperature using III–V Nitride based triangular microcantilever heater. *Sens. Actuators B* **222,** 459–467 (2016).
